# What in the World Is Collective Responsibility?

**DOI:** 10.1111/1746-8361.12228

**Published:** 2018-08-24

**Authors:** Alberto Giubilini, Neil Levy

**Affiliations:** ^1^ Oxford Martin School and Wellcome Centre for Ethics and Humanities University of Oxford; ^2^ Oxford Martin School and Uehiro Centre for Practical Ethics University of Oxford and Macquarie University

## Abstract

In this paper we analyse the notion of collective responsibility and the criteria for its application to different types of groups. We argue that most of the ways in which the notion of collective responsibility has been attributed to different types of groups actually refer to a form of responsibility that is not genuinely collective, but that boils down to some form of individual responsibility. We identify an intrinsically collective kind of responsibility and argue that it can be attributed to only one kind of group. We begin by setting two necessary and sufficient conditions for attribution of genuinely collective moral responsibility, asking whether these two conditions are satisfied in the case of different types of groups that have been taken to be bearers of moral responsibility: organized groups, groups with internal bonds of solidarity, groups that program individuals to act in a certain way, random collections of individuals, and individuals engaging in joint actions. Contrary to what various authors have maintained, we argue that only in the case of individuals engaging in joint actions is attribution of a genuinely collective form of moral responsibility warranted, i.e. only groups engaging in joint action satisfy the two conditions for attribution of genuinely collective moral responsibility.

## 1 Introduction

Is there such a thing as “collective responsibility”? For example, can a corporation, a state, a mob, a social movement, a random collection of individuals be *collectively* morally responsible for things like an environmental disaster, a bad policy, an act of violence, a form of discrimination, or the failure to realize herd immunity or to help people in need? Or is it only individual members of these collectives who are morally responsible for such states of affairs? The answers to such questions depend on what it means to attribute to an entity a form of *moral* responsibility (as opposed to merely causal responsibility) and on what it means to say that the responsibility in question is *collective* (as opposed to individual). The focus of this paper will be the latter question. Whereas debates over the moral responsibility of collectives have largely focused on the question whether collectives can be morally responsible, we want to bypass this debate in order to ask a different question: *given that* collectives can be morally responsible, is there a kind of moral responsibility that applies to them that is *intrinsically* collective? We answer in the affirmative: if collectives can be morally responsible, there is a *deeply collective* form of (retrospective) moral responsibility.

We will argue that most of the ways in which the notion of collective responsibility has been used in the debate refer to a form of responsibility that is not genuinely collective, but that boils down to some form of individual responsibility attributed to collectives. We argue that only individuals engaging in *joint actions* satisfy what we take to be the two necessary and sufficient conditions for attribution of the *genuinely* collective form of moral responsibility that we present in this paper. Thus, the aim of this paper is to set out the conditions for genuinely collective moral responsibility and to show that only one particular type of collective satisfies them.

Before we turn to delineating the two necessary conditions that groups must satisfy in order to be justifiably attributed this intrinsically collective kind of moral responsibility, we must say a few words about what we mean by ‘moral responsibility’. This is important, not only because there are multiple senses of the phrase (Smiley 1992; Fischer and Tognazzini 2011), and different groups might plausibly be morally responsible in some of these senses and not others, but because we need to allay worries that the kind of intrinsically collective moral responsibility we identify is not a kind of moral responsibility at all.

We will use the phrase ‘moral responsibility’ in what we take to be its central sense, often called the ‘basic desert’ sense (Pereboom 2001; 2013). In this sense, to say that someone is morally responsible for a certain outcome is to say that she is the appropriate target of reactive attitudes like indignation, resentment or gratitude (Strawson 1962) just in virtue of realizing that outcome or for failing to realize a desirable outcome. Sometimes attribution of moral responsibility in this sense is cashed out in terms of attribution of moral *blameworthiness* (Smiley 2010). It is this sense of responsibility that is most often adopted in discussion about collective moral responsibility – with some important exceptions (e.g. Smiley 2014) 1In recent years, interest in forward‐looking collective responsibility has grown, as demonstrated, for example, by the 2014 issue of *Midwest Studies in Philosophy* (38, 1) dedicated to it. Sometimes prospective responsibility takes the form of “remedial responsibility”, i.e. responsibility to remedy a situation for which a collective might be held causally or morally responsible, or which a collective has the capacity to remedy even if it is not morally or causally responsible for it (Miller 2001). – and this is also the notion of collective responsibility we will consider in this paper. From this point on, therefore, whenever we refer to ‘collective responsibility’, it is the basic desert sense we have in mind: when we say that some groups can be morally responsible in an intrinsically collective way, we mean that they can be deserving of blame, praise or sanction, entirely in virtue of past actions or omissions, in a way that is intrinsically collective. Intrinsically collective moral responsibility, so conceived, is a subset of the moral responsibility appropriately attributed to groups. Most philosophers have been concerned with whether groups can be morally responsible (usually, but not always, in the basic desert sense), not whether the kind of moral responsibility appropriately attributed to them is intrinsically collective. This focus is understandable: the central normative issues up for grabs (Should groups be blamed? Are they appropriately sanctioned? Should the individuals who constitute them feel guilt? and so on) do not seem to depend on whether the moral responsibility at issue is intrinsically collective. It may be, however, that intrinsically collective moral responsibility has somewhat different implications for how we should respond to groups and/or their constituent members. Perhaps (for instance), when moral responsibility is intrinsically collective, blame attaches less, or not at all, to individuals. Identifying this intrinsically collective moral responsibility is the first step towards answering these questions. If such a kind of moral responsibility exists, it is genuine moral responsibility (by satisfying the conditions required by a basic desert conception), but a metaphysically interesting subset of such moral responsibility, insofar as it is intrinsically collective.

We now turn to the aspect of collective responsibility in which we are interested in here, i.e. the ‘collective’ part of the notion of ‘collective responsibility’. We accept, of course, that there are multiple ways of legitimately using words like ‘collective.’ We might justifiably say that a group is collectively responsible just in case it is a collective, in some sense, and it is appropriately held responsible. Here we are after something deeper, something more intrinsically collective *about* the moral responsibility attributed. Responsibility is collective in our sense if two conditions are satisfied. First, it must be the case that attributions of collective responsibility (e.g. “Germany was collectively responsible for the Holocaust”; “naturopaths are collectively responsible for the loss of herd immunity”; “Exxon is collectively responsible for climate change”) are not metaphorical, or best explained as shorthand for a more complex claim about attributions of an individual form of responsibility (like “a large number of ordinary Germans are individually responsible for the Holocaust” or “senior executives at Exxon are responsible for climate change”). Second, the kind of collective responsibility attributed to the group must not be individual responsibility scaled up, applying to a collective because it satisfies one or more of the metaphysical conditions required for moral responsibility. If it exists, it is a sui generis kind of responsibility, which in this paper we will call *genuinely* collective responsibility. As mentioned above, there are two conditions that collective responsibility must satisfy in order to be *genuinely* collective: in the next section we introduce these two conditions.

## 2 Two conditions for genuinely collective moral responsibility

For attribution of genuinely collective moral responsibility, the responsibility in question must not be reducible to a mere aggregation of individual responsibilities. “Methodological individualists” are sceptical that this condition is ever satisfied by collectives (Lewis 1948; Benjamin 1976; Narveson 2002). 2In principle, methodological individualism is (as the name suggests) a methodological principle alone. It does not commit a proponent to the view that collectives are ontologically reducible to individuals. However, in practice, methodological individualists hold the associated ontological view as well. We therefore follow writers like Lukes (1968) in interpreting methodological individualists as holding the corresponding ontological thesis. For J. Angelo Corlett, for example, propositions like “the Exxon Corporation is responsible for an oil spill” are “reducible and redescribable in terms of the responsibility and punishability of certain members of the Board of Directors, and other higher‐level managers, who served Exxon Corporation at the time of the decisions made which ‘caused’ the incident” (Corlett 2001a, 103). Thus, a first condition for attribution of genuinely collective moral responsibility is that methodological individualism be false. But what characteristics must a group possess for methodological individualism to be false? And how can this condition be more precisely formulated?

Now, the question we are interested in, namely the question as to what types of groups can be held *collectively* responsible – call it the “assignment of blame question” – is distinct from the question as to whether certain actions can be considered actions *of the group* as an entity not reducible to an aggregation of individuals – call it the “ascription question”. However, the assignment of blame question and ascription question are closely related. The link between the two lies in the fact that attribution of responsibility to a collective requires that there is a collective agent that is not identified simply as an aggregation of individual agents – in which case the responsibility in question would only be the responsibility of each individual. We can refer to this characteristic by saying that the collective agent must have its own *identity*, whereby ‘identity’ we will simply mean the ontological status of an entity, e.g. the identification of a certain entity as either an aggregation of individuals or as a unified agent itself. Thus, to say that a collective agent must have its own identity is to say that it must not exist as a mere aggregation of individual identities, but that it must have its own distinctive ontological status. Without satisfying this condition, it is hard to see how a collective can be the subject of a type of moral responsibility that does not boil down to the responsibility of its individual members.

Moreover, besides its own identity, the collective entity must have, *qua* collective, the capacity to *intend* to bring about or to prevent the morally relevant outcome, or at least to reasonably foresee that that outcome would or would not occur as a consequence of the agent's behaviour. Accordingly, having a certain intention or being capable of foreseeing certain outcomes – here we will use the term “intention” broadly to refer to both aspects – are necessary conditions for attribution of moral responsibility. The intention in question needs to be describable as a genuinely collective intention, rather than as merely the collection of the individual intentions of its members, in order for it to count as a reason for attribution of genuinely collective moral responsibility. Further, attribution of responsibility requires that the agent *can act* as a collective: we must be able to hold a collective responsible for actions that are actions of the collective and not simply an aggregation of the actions of individual members. Thus, the first condition for attribution of genuinely collective moral responsibility can be reformulated as follows: for a collective to be genuinely collectively responsible in a way that does not boil down to a mere aggregation of individual responsibilities, it is essential that the collective possesses *its own* identity and *its own* intentions and that it perform *its own* actions. It is only when we answer the ascription question by saying that the identity, intentions and actions at stake are the identity, intentions and actions *of a group* that we can answer the “assignment of blame question” by saying that the group as a collective is morally responsible for the action or its outcomes. An entity cannot be held morally responsible if *it* doesn’t have an identity and an intention or if *it* doesn’t perform the action for which it is held responsible.

Many contributors to debates over the moral responsibility of collectives have explicitly rejected this condition (see, for example, Wringe 2016). It is important to recognize that we need not take issue with these philosophers. Our aim is different from theirs: they are concerned with the question whether groups can be morally responsible, in the basic desert sense or in a forwards‐looking sense, not with whether their responsibility is intrinsically collective. Our first condition is a necessary condition of intrinsically collective moral responsibility: it may not be a necessary condition of collective moral responsibility.

A second condition for attribution of genuinely collective moral responsibility is that a collective that has its own identity, its own intentions and performs its own action is appropriately held responsible in a way that accounts for its collective character, i.e. for the fact that the collective is constituted by a number of individuals. In other words, for its responsibility to be genuinely collective, rather than individual responsibility scaled up, this kind of responsibility must be attributed to the collective as a composite, and not an individual, entity: it must not be a form of individual responsibility that happens to apply to collectives. Thus, satisfaction of the second condition for attribution of genuinely collective moral responsibility requires that the characteristics that are relevant for attribution of moral responsibility, namely the identity, intentions and actions of the collective, be the product, or exist in virtue of, the identity, intentions and actions of its individual members. The reason why this is a necessary condition for ascription of genuinely collective moral responsibility is that we need a criterion to distinguish groups that can be considered morally responsible in the same sense as individuals are – and in the same sense in which, for example, corporations can be considered persons from a juridical perspective – from groups that can be considered morally responsible in such a way that their being *collective* makes an important difference to whether moral responsibility can be attributed to them as composite entities.

Identifying collectives that may be genuinely collectively responsible is a difficult task because, at a first glance, the two conditions for attribution of genuinely collective moral responsibility seem to contradict one another: according to the ascription condition, the collective must not be reducible to an aggregation of individuals in the morally relevant aspects (identity, intentions and actions), but at the same time, according to the second condition, the same collective must be held responsible in a way that accounts for the fact that it is composed by individuals. However, these two conditions do not contradict one another *necessarily*: as we have mentioned above and as we will argue more extensively in section 3.5, there is one type of collective that meets both conditions, and therefore one – and only one – type of collective that can be the bearer of a type of responsibility that is not merely a form of individual responsibility.

Now, the two conditions we have put forward are individually necessary, but they are not jointly sufficient for attribution of genuinely collective moral responsibility. Clearly, collectives need other properties in order to be proper subjects of some form of moral responsibility. For example, one might say that collectives need to be able to make moral judgments or act on moral considerations. However, we will not consider these further conditions here. As we have said above, demonstrating that collectives satisfy these further conditions requires that they satisfy demanding metaphysical conditions, which we do not have space to discuss here. There is already a rich literature on whether collectives can be morally responsibile, in the basic desert sense. For the purposes of this paper, we assume that this literature succeeds in showing that groups can be morally responsible in this sense. Given that this is the case, we are concerned with the further question whether this responsibility is intrinsically collective.

As we are going to argue, whether collectives can be considered morally responsible in a genuinely collective sense depends on the type of collective considered. Let's examine, then, in the next section, how the notion of collective responsibility could be, and has been, attributed to different types of collectives, and whether this attribution is warranted in light of the two conditions we have just introduced. In subsections 3.1–3.4 we will argue that, contrary to what other authors have maintained, such attribution is not warranted for certain types of collectives. In subsection 3.5, we will discuss the only type of collective that we think can be attributed genuinely collective moral responsibility.

## 3 Types of collective moral responsibility

We can identify five main types of collectives that have been considered bearers of moral responsibility. These collectives are: (1) organized groups, to which some have attributed *corporate* responsibility (and which we will discuss in section 3.1); (2) groups identified by bonds of mutual solidarity among their members or by individuals’ identification with the group, and whose members might be attributed *vicarious* liability (section 3.2); (3) groups that ‘program’ individuals to act in a certain way, and to which some attribute a *restricted* form of collective responsibility (section 3.3); (4) random collections of individuals, which have been attributed a *distributive* form of collective responsibility (section 3.4); and (5) groups that can be identified by the fact that their members perform joint actions, which, as we will claim, can be attributed *genuinely collective* moral responsibility (section 3.5).

It is important to point out that these are not mutually exclusive categories: a collection of individuals might fall into more than one. For example, members of a corporation can act in virtue of internal bonds of solidarity, or members of a mob that ‘programs’ individuals to act in a certain way can engage in joint actions. We will examine these groups separately for conceptual clarity, but this does not exclude the possibility that groups might be attributed different types of responsibility in virtue of properties other than those we focus on each time.

### 3.1 Responsibility of organized groups

When a collective is morally responsible, in either the scaled‐up sense(s) or the genuinely collective sense, its responsibility is not reducible to a mere aggregation of individual responsibilities and therefore does not necessarily have implications for attribution of moral responsibility to particular individuals. It is therefore conceptually possible that collectives bear moral responsibility for a certain outcome even if not all individual members are, all things considered, morally responsible for that outcome or have the corresponding moral obligations (Cooper 1972; Copp 2007, 374).

Collective responsibility is therefore most straightforwardly attributed to groups that exist as independent entities characterized by distinctive structural features – e.g. internal constitutions, decision‐making procedures – which guarantee that their identity as a group survives changes of individual membership (List and Pettit 2011, 31). In other words, the identities of such groups are “not exhausted by the conjunction of the identities of the persons in the organization” (French 1984, 13). Peter French (1984, 13) and Angelo Corlett (2001b, 573) call these groups “conglomerates”, and Christian List and Philip Pettit call them “corporate agents” (Pettit 2007; List and Pettit 2011). Examples of such groups include institutions such as states and business corporations.

On some accounts, these types of groups can be held collectively morally responsible even if they lack the capacity to act on the basis of intentions, as is instead required by Kantian notions of blameworthiness (Smiley 2010). According to these accounts, “the ability to act and to intend is not a condition of moral responsibility *per se*” (Smiley 2010, 171; for a criticism of this type of view, see Corlett 2001b). More particularly, on the account proposed by Smiley, in order for such groups to be held responsible it is sufficient that they can *create* or *produce* harms in a way in which individuals alone cannot and which presupposes control by the group over the harm's occurrence (Smiley 1992, 196). For example, a corporation that causes an environmental disaster can be held morally responsible for it merely in virtue of producing the harm even in the absence of group agency. On this view, creating or producing harm, which only requires the causal power to bring about the harm, is sufficient for moral responsibility; the more demanding characteristics required for agency are not required.

On other accounts, organized groups can be considered analogous to individual agents for the purpose of attribution of moral responsibility, in virtue of internal rules and procedures that determine their decision making (French 1984; Corlett 2001b; Pettit 2007; List and Pettit 2011). Such internal rules qualify them as “secondary agents” (Copp 1979), i.e. agents “for whom another acts according to a legal or a moral rule system, intentionally” (Corlett 2001b, 578). As put by Pettit, corporate agents “operate through their members in such a way that they simulate the performance of individual agents” (Pettit 2007, 172). On these accounts, such groups are ascribed representational states (e.g. beliefs), motivational states (e.g. desires), and the capacity to process and act on the basis of such states independently of the beliefs and the desires of individual members (French 1984; Pettit 2007; List and Pettit 2011, 20–24; Björnsson and Hess 2017). In other words, such groups have been taken to display “patterns of attitudinal and behavioural rationality” (Pettit 2007, 178) that explain their behaviour and that therefore warrant attribution of agency and of moral responsibility: such agents are *attributed* the capacity to act for reasons. According to Corlett, “there seems to be no conceptual barrier to constructing secondary agents as intentional ones” (Corlett 2001b, 578) – although Corlett also thinks that in practice it is very rare that conglomerates act as intentional agents, since most of the time the actions of conglomerates are actually caused by desires or beliefs of powerful individual members of the conglomerate (Corlett 2001b, 579).

Thus, for example, when a group such as a corporation is a ‘secondary agent’, its decision to adopt a certain policy that involves the release of polluting substances might be explained by the corporation's desire to realize a profit and its belief that a policy that involves the release of polluting substances is the most effective way to realize the profit. Similarly, a state's decision not to introduce a compulsory vaccination policy may be explained by its belief that compulsory vaccination is not necessary for realizing herd immunity, or by its belief that compulsory vaccination violates some individual right as established by the state's constitution or legal system.

It is important to note that, if we accept that such groups can be attributed desires, beliefs and intentions, then group agents like these can be attributed identity, intentions and actions that do not boil down to a mere aggregation of the identities, intentions and actions of their individual members. Therefore, such groups meet the first condition of attribution of collective responsibility: the responsibility in question is not just a sum of individual responsibilities. According to Pettit, “a group agent can act to bring about some harm, or indeed some good, without any of its members being fully fit to be held responsible for his or her contribution to that result” (Pettit 2007, 196), for example because they are blamelessly ignorant of a collective harm done, or they reasonably believe that they would not make any difference to such harm, or are under pressure to act (Pettit 2007, 196). As put by Steven Sverdlik, in the case of corporate agents “it is an open question whether the individuals in the group are also responsible for the outcome or whether the group as such is alone responsible” (Sverdlik 1987, 62).

However, even if we accept all these premises, such groups fail to meet the second condition for attribution of genuinely collective moral responsibility, namely that the responsibility in question should not be simply a kind of ordinary individual responsibility where the individual happens to be a composite entity. The responsibility in question is individual in nature, i.e. attribution of responsibility does not depend on the composite nature of the collective in question. The identity, intentions and actions of the group do not necessarily depend on, or are not necessarily the product of, the identities, intentions and actions of individual members (although they might be, in cases in which such individual members of a corporation engage in joint actions, as we will see below). Thus, group intentions (or actions or identities) that exist independently of the existence of intentions (or actions or identities) of individual agents do not suffice for attribution of genuinely collective moral responsibility because the group intentions (or actions or identities) do not depend on the *collective* character of the group. The group is rather an individual entity with its own intentions and performing its own actions, which warrants attribution of only an individual type of responsibility. In fact, in order to stress the fact that the group agent bears moral responsibility in a way that is independent from the composite nature of the group, the notion of “corporate responsibility” (French 1984; Sverdlik 1987; Pettit 2007; List and Pettit 2011, 167–169; Sepinwall 2016), rather than that of “collective responsibility”, is often adopted to refer to the responsibility of organized groups. In fact, because corporate responsibility is independent of attribution of responsibility to individual members of the group, some do not consider it an instance of *collective* responsibility at all (List and Pettit 2011, 167–169; Sverdlik 1987): in contrast to what occurs in cases of collective responsibility, “with corporate responsibility the group is treated as a being distinct from its members and responsibility for wrongdoing is attributed to it” (Sverdlik 1987, 62). As stated above, this does not entail that individuals cannot be held morally responsible for making it possible that the group causes a certain morally relevant outcome – perhaps in most cases individuals would be individually morally responsible for this. But this kind of group is held responsible as an independent and unified agent, rather than as an entity formed by a number of individuals. For this reason, the responsibility in question is better understood as a type of individual responsibility scaled up and attributed to a peculiar type of agent, rather than as a form of *genuinely* collective responsibility.

Now, one might object here that the actions of an organized group necessarily depend on the actions of their individual agents, and therefore that in an important sense organized groups do meet the second condition for attribution of genuinely collective moral responsibility. 3We are grateful to an anonymous reviewer for raising this objection. This is an important observation that needs to be addressed. It is true that organized groups’ actions are the product of individual actions, but this description needs to be qualified. Organized groups’ actions are the product of individual actions in the obvious sense that, without individuals acting in a certain way, there would be no group action. However, this obvious fact is not enough to satisfy the second condition for attribution of genuinely collective moral responsibility, i.e. that, to repeat what we have said above, “responsibility must be attributed to the collective as a composite, and not an individual, entity: it must not be a form of individual responsibility that happens to apply to collectives”. In the case of organized groups’ actions, the responsibility and the action remain the responsibility and the action of the group as a *sui generis* individual because individual members always act *on behalf* of the group, i.e. as mere instruments necessary for carrying out the group's venture. For example, if the CEOs or the board of directors of a corporation decide to implement a certain policy, they do so because they want the corporation to act in a certain way and therefore they make decisions and act in such a way that the company can carry out its actions; but in principle – and differently from the case of joint actions we will discuss in section 3.5 – one individual, say the CEO, could alone generate the organized group's action as long as she acts on behalf of the group, for example by signing a form that authorizes the release of polluting waste into a river: this would be the action of the organized group even if it is caused by just one individual (which is not the case for joint actions we will discuss in section 3.5). So, the group need not be a composite entity in order to act as a *sui generis* individual; the only thing that matters for attribution of agency to the organized group is that at least one individual member of the group acts *on behalf* of the group. In fact, it is the group's structure and mission that set the responsibilities and therefore the expected actions of CEOs or of any other members, rather than the other way around; in other words, the group pre‐exists individuals, and its existence makes it possible and explains the actions of the individuals who act on its behalf. Therefore, the actions of an organized group are the *product* of individual actions in a sense that is not sufficiently strong to justify attributing to the group a form of agency, and therefore a form of moral responsibility, that is genuinely collective rather than individual scaled up, as the second condition we set above would require.

### 3.2 Responsibility of groups with internal solidarity

Sometimes collectives are held morally responsible for actions or outcomes in the sense that each individual member of the group is held responsible for actions performed by only some of them. This happens when individuals identify or have solidarity with the actors, regardless of whether they themselves were actors (Feinberg 1968; McGary 1986; May 1987; Moody‐Adams 1994; Radzik 2001; Silver 2002). In cases like this, we may say that the group acted, while recognizing that not every member of the group acted. For example, one might say that the Germans were in this sense responsible for the Nazi crimes – unless they dissociated themselves from the Nazi practices. The behaviour of some members is attributed to all, because non‐actors and actors alike have solidarity with each other. According to Feinberg, a group has solidarity when its members have “mutual interests, bonds of affection, and a ‘common lot’”, i.e. their goods and harms are collective and indivisible (Feinberg 1968, 677). Examples of such groups typically include nations, social movements or racial groups, but organized groups such as states or corporations might also be considered collectively responsible in this sense, to the extent that members endorse (or fail to repudiate) the group's behaviour. These groups might be held collectively morally responsible inasmuch as, through relationships of solidarity among members, the “contributory fault” (Feinberg 1968) of any one individual, i.e. the contribution of any one individual to the morally relevant outcome collectively brought about, is transferred to all the members who identify with the group, or have solidarity with the group, or do not sufficiently dissociate themselves from a certain practice of the group (McGary 1986).

This form of responsibility is an instance of what Joel Feinberg called “vicarious liability”, which according to Feinberg is a form of “collective liability” applied to organized groups (Feinberg 1968, 677). As put by Feinberg, “[w] hen the whole group is held responsible for the actions of one or some of its members, then from the point of view of any given ‘responsible’ individual, *his* liability in most cases will be vicarious” (Feinberg 1968, 677). On some accounts, solidarity with a group renders individuals responsible even for the wrongdoing perpetrated by previous generations, especially when there is some form of cross‐generational identification (Silver 2002; Abdel‐Nour 2003). Escaping such vicarious liability will require, at minimum, repudiating the wrongdoing and perhaps rejecting any benefits flowing from it (McGary 1986, 163).

There are, however, grounds for denying that vicarious liability is a form of genuinely collective responsibility. It seems that the responsibility here applies to individuals who form bonds of solidarity or identify with the collective, and not to the collective itself; talk of collective responsibility in such cases seems to be merely metaphorical. While the bonds of solidarity might partially constitute the collective identity, it is not the collective that is held responsible. Rather, it is each member of the collective who is, individually, responsible. Thus, vicarious liability fails to meet the first condition for attribution of genuinely collective moral responsibility: the identities, intentions and actions involved are merely the identities, intentions and actions of the individuals who form bonds of solidarity with each other; accordingly, all the responsibility involved seems to be the aggregation of individual responsibilities for forming bonds of solidarity with actors engaging in wrongful actions, or for not dissociating oneself from such actions. 4A reviewer for this journal points out that solidarity groups can engage in actions with essentially relational explanations, and such groups would satisfy our first condition. Indeed, the reviewer thinks that such groups may satisfy this condition better than those that engage in joint action, because their goal may have collective content (e.g. “that we perpetuate our culture”). It seems to us that the reviewer is correct that solidarity groups can engage in actions that satisfy our condition. However, when solidarity groups satisfy this condition, we claim, it is not in virtue of being solidarity groups that they do so (though being solidarity groups may help to explain their satisfaction of this condition). Rather, it is in virtue of engaging in joint action that such groups satisfy our first condition. We thank the reviewer for highlighting the importance of goals with collective contents. We take this to be a friendly amendment, more than an objection: when groups have such collective goals as well as collective intentions, they may plausibly be said to engage in more deeply joint action than when they do not. However, having such collective goals does not seem a requirement for intrinsically collective moral responsibility.


### 3.3 Responsibility of “programming” groups

In a third sense, groups may be held to be collectively responsible on the grounds that only the existence of the collective can explain the actions of individuals and their outcomes, and this entails that the group is morally responsible for such actions and outcomes (Pettit 2007, 192; Shockley 2007) even without group agency. This occurs when individuals act *qua* group members, i.e. in ways in which they would not have acted had they not been members of the group. For example, a member of the Ku Klux Klan who participates in violence against a black person might be doing so because she knows that her participation is what is required of KKK members. On this account, the KKK might be held collectively morally responsible for the violent behaviour of its members. In this case, we are dealing with a case of ascription of collective responsibility in the absence of collective agency and collective intentionality, and therefore, following Kenneth Shockley, we might consider the responsibility in question a “restricted” form of collective moral responsibility (Shockley 2007, 442). Such groups nevertheless meet the conditions set down by Smiley's account of collective responsibility discussed above: the group can be held responsible for an outcome because the group *creates* or *produces* harms in a way in which an individual cannot, even in the absence of group intentionality or agency. On Smiley's account, “creating” or “producing” harm is taken to include “enabling or leading group members to perform harmful actions” (Smiley 1992, 196), which determines the particular type of collective responsibility without collective agency we discuss in this section.

This understanding of collective responsibility is sometimes applied to those groups that can be said to “program” individual members to act in certain ways (Pettit 2007, 192; Shockley 2007), such as mobs (Shockley 2007) or corporate agents (Shockley 2007, 449; Smiley 2010, 197). That a group “programs” individuals’ behaviours – and is therefore held by some to be morally responsible for these behaviours – means that the group plays an ineliminable explanatory role in an account not only of why individuals act in a certain way (Smiley 2010, 197), but also of how a resulting morally relevant outcome is brought about (Shockley 2007). In such cases, individuals’ behaviours typically produce effects they would not have produced had they not acted *qua* group members. For example, the existence of a violent mob or of a group like the Ku Klux Klan explains both why individual members of the mob or of the Ku Klux Klan engage in acts of violence and why individual actions together produce the collective outcome that they do, e.g. a climate of lawlessness and/or fear. Neither the individual actions nor the outcome would have occurred had the mob or the Ku Klux Klan not existed, and had individuals acted in isolation. It is important to stress that the outcome itself may be different in kind from the one that might be produced by individuals acting in isolation, due to its collective production: for example, the fear generated by a mob is different in kind from the fear generated by any single act of violence of its members (Shockley 2007, 451). This is why it is sometimes implied that there is a type of moral responsibility that is ascribable only to the group. As put by Shockley, “we can (…) attribute moral blame to collectives that program individuals to perform blameworthy acts where those acts, on aggregation, constitute a different form of harm” (Shockley 2007, 451). Thus, we can say that the fact that the group causes individuals to act in certain ways and the fact that the harm produced could not have been produced by an individual acting in isolation – i.e. that the group “programs” individual behaviours – are taken to be jointly necessary and sufficient conditions for attribution of moral responsibility to the group in question.

However, it is not clear why the responsibility attributed to the group could not be considered a form of merely *causal*, rather than moral responsibility (Benjamin 1976, 101–102), even if we adopted a form of merely “restricted” moral responsibility. In fact, what Shockley calls “restricted” moral responsibility seems not to be a form of “moral” responsibility at all, since the type of responsibility involved does not require that the group in question possesses those properties that are relevant for ascription of moral responsibility – i.e. its own identity, its own intentions and its own agency. Let's see in more detail.

Individual members of the group can legitimately be considered morally responsible to the extent that they allow the group to influence their individual behaviours. However, as far as the “programming” is concerned, what the group is responsible for is merely its *explanatory* role – what Shockley calls the “explanatory significance of collectives” (Shockley 2007, 444) – in an account of how individuals came to act in certain ways and how they together produced a certain outcome that is qualitatively different from the outcome they would have otherwise produced through their individual actions. But this explanatory role seems to warrant only attribution of causal responsibility to the group: in order to be able to “program” individuals to act in a certain way and to fulfil its explanatory role in the account of why individuals acted in that way, the group need not have morally relevant features – i.e. features that warrant attribution of moral responsibility – such as its own identity, intentions and agency. Accordingly, we have no reason to postulate that the responsibility for programming individual actions is “moral” responsibility. And since the production of the collective outcome and the individual behaviours are all that a programming group is held responsible for, all the responsibility we can attribute to the group is merely causal, not moral responsibility. In other words, “programming” is a matter of causing certain individual behaviours or of causing the individual attitudes that might result in certain individual behaviours.

Note that this account of programming groups does not entail that the individual members of a programming group *necessarily* act together with other group members, i.e. that they perform a “joint action” of the kind we will describe in section 3.5 (and which are based on Bratman's account of shared intention). In the case of programming collectives, each individual acts individually – by performing her “contributory action” – in the pursuit of a common end or of an end that is complementary to the ends of other group members, without necessarily cooperating explicitly or intentionally with other group members (Shockley 2007, 446), as is instead the case with people engaging in joint action. Think, for example, of the difference between individual members of a mob (i.e. a programming group) each one engaging in acts of violence and individuals who act together to lift a heavy table (i.e. a group engaged in a joint action). Each individual in the mob might individually – as opposed to through joint action – engage in acts of violence in order to pursue the common end of generating a climate of fear or lawlessness (Shockley 2007, 448). It is however possible, and indeed likely, that individuals programmed by groups to act in a certain way thereby engage in joint actions; therefore, although there is a *conceptual* difference between programming groups and groups engaged in joint action for what concerns the collective character of the action involved, *in practice* the two types of group can and often will overlap. Indeed, as a rule of thumb, we can say that that the practical overlapping between programming groups and groups engaged in joint actions is the more unlikely, the more the programming group approximates in size large‐scale venture groups (Shapiro 2014). According to Scott Shapiro, large‐scale venture groups cannot be said to engage in joint activity because large numbers of individuals are unlikely to be able to share the necessary commitment required for joint action; in Shapiro's words, “in any large‐scale activity, there are bound to be participants that intentionally contribute to the group effort but are not committed to the success of the group venture” (Shapiro 2014), and as we shall see in section 3.5, some form of commitment to the success of the group venture is necessary to qualify that venture as a form of “joint action”. Thus, the larger the programming group is, the more unlikely it is that it will form a group engaged in a joint action, and therefore that the conceptual difference between the two will translate into a practical overlapping

In the case here under examination, then, it seems that groups identified by their programming individual behaviours do not satisfy the first condition for attribution of moral responsibility to collectives: the identities, intentions and actions involved are only the identities, intentions and actions of individual members; accordingly, all the *moral* – as opposed to causal – responsibility that there is in such cases is the moral responsibility of individual members for their individual behaviours, even if this behaviour can be *explained* by the existence of the collective. Such moral responsibility of individuals cannot translate into a genuinely collective form of moral responsibility attributed to the group, because the identity, the intentions and the actions involved, i.e. the morally relevant aspects that warrant attribution of moral responsibility, are just the aggregation of the identities, the intentions and the actions of its individual members: the programming does not happen because the group is a collective agent, with its own collective intention performing a collective action, but it only happens because the group has the merely causal power to ensure that individuals engage in certain behaviours.

Perhaps some people are tempted to ascribe moral responsibility to the group because the group's existence might reduce the moral responsibility of its members (for instance by cultivating an atmosphere of lawlessness that makes better behaviour more difficult). But it is a mistake to think that a certain quantum of moral responsibility must be preserved: the group may reduce the moral responsibility of its members without it being morally responsible itself.

### 3.4 Responsibility of random collections

In a fourth sense of “collective responsibility”, a collection of individuals might be considered morally responsible on the grounds that only the coordinated or the aggregated individual behaviours of a large enough number of individuals, and no single individual behaviour, can cause or prevent a certain morally relevant outcome (Held 1970; Sverdlik 1987, 63; Sadler 2007). For example, one might say that individuals who could, but fail to, coordinate in order to remove the beams that keep a man trapped under a collapsed building are collectively morally responsible for the death of the man or for failing to take action to save the man (Held 1970); or that those who drive every day are collectively morally responsible for global warming (Sadler 2007); or that individuals who cooperate to drag a car into a lake are collectively responsible for the resulting damage to the car (Mellema 2006). The first two examples involve random collections, while the third example involves a collection identified by the performance of a joint action. We will discuss the responsibility of random collections in this section and the responsibility of individuals engaging in joint actions in the next.

A random collection might be defined as “a set of persons distinguishable by some characteristics from the set of all persons, but lacking a decision method for taking action that is distinguishable from such decision methods, if there are any, as are possessed by all persons” (Held 1970, 471). Larry May calls these collections “loosely structured groups” (May 1990). For the purpose of attribution of responsibility to it, we can identify a relevant random collection of individuals by the fact that (1) each of them is in a position in which they can reasonably contribute to the realization or the prevention of a certain morally relevant outcome, that (2) only together do they have the causal power to realize or prevent a certain morally relevant outcome, and that (3) there is no other feature they share (e.g. mutual solidarity, or being “programmed” by the group) that could account for their inclusion in a certain group.

As the examples we have used at the beginning of this section suggest, there are two types of random collections that are candidates for attribution of moral responsibility: (1) random collections identified by collective inaction (Held 1970; May 1990) and (2) random collections that are held responsible for the aggregate behaviours of individuals (Sadler 2007). Let's consider them in order.

(1) *Random collections and collective inaction*. Sometimes random collections are identified by the fact that their individual members fail to coordinate and take action when they plausibly should. Consider the following example due to Virginia Held (1970). Three pedestrians notice a man trapped under a collapsed building; they could save the man by together removing various beams that keep him trapped and which can only be moved by all three acting together, but they can’t agree as to which beam to remove first; as a result, the man dies. Held argues that in such a case, since it is obvious to any reasonable person what the individuals should have done to prevent the undesirable outcome (Held 1970, 478) – namely coordinate with others to perform a certain joint action – the *random collection* formed by the individuals who were in a position to make their contribution is morally responsible “for failing to turn itself into an organized group capable of taking action requiring a decision” (Held 1970, 479). In Larry May's words, the collection is morally responsible for its “collective inaction” (May 1990), and in Tracy Isaacs's words, the collective might be held responsible because, even if it is a merely random collection, it is a “putative,” or “potential” group agent that fails to meet a “putative collective obligation” (Isaacs 2011, especially chapter 5; 2014).

This form of responsibility of random collections is taken by Held to be “distributive”, by which Held means that, “if random collection R is morally responsible for the failure to do A, then every member of R is morally responsible for the failure to do A, although, perhaps, in significantly different proportions” (Held 1970, 480). Along similar lines, May thinks that, in the case of “collective inaction”, responsibility is shared among individuals, but he adds that it should not necessarily be apportioned equally; for example, some members may play a more significant role than others in shaping the intentions to act of other members of the collective, and therefore might have a greater responsibility for the group's inaction (May 1990, 274). Finally, according to Isaacs, “once it is clear what is required of the collective, the collective obligation has a derivative impact on the obligations of individuals” (Isaacs 2011, 150).

However, if the responsibility at issue is collective in a distributive sense or in a way that shapes individual responsibilities, it is not clear how the statement that a random collection is *collectively* morally responsible is supposed to be different from the simple statement that each individual is morally responsible for the failure to contribute to a certain desirable outcome. Indeed, if some individual did (try to) contribute to the desirable outcome, e.g. tried to cooperate with others while others were not responsive to the request for cooperation, then this individual, by definition, would not be part of the random collection characterized by collective inaction (Bates 1971, 348). Thus, the collective is only formed by those individuals who are, *individually*, morally responsible for failing to make their contribution to the performance of a joint action. But, since they constitute a merely *random* collection, there is no form of cooperation or any other type of relationship between these individuals that determines the existence of a collective super‐entity to which some form of moral responsibility, different from the responsibility attributed to each individual member, could be attributed. Each individual is morally responsible, but there is no entity to which we can attribute some form of responsibility different from the responsibility attributed to individuals. Thus, collectives identified by collective inaction also fail to meet the first condition for attribution of genuinely collective moral responsibility: the identities, intentions and (in) actions of the collective that explain the failure to realize the desirable collective outcome are nothing more than the aggregation of the individual identities, intentions and (in) actions of members of the collective who failed to make their contribution to the collective outcome. 5A reviewer for this journal suggests that responsibility for failures to perform joint actions requires nothing more than individuals standing in normative relations; isn’t this a kind of collective responsibility, however, of the kind that Miller (2006) captures? We agree with both points: we maintain, however, that responsibility for such a failure is not an intrinsically collective kind of responsibility. There is an asymmetry here: *had* the group acted, it would have been responsible in our deeply collective manner, but its responsibility for its failure to act is of a different kind (though not necessarily any less serious). We do not think that this is problematic: acting together requires a kind of coordination and shared intentions, whereas failing to act together can be done any old how.


(2) *Random collections and aggregate individual behaviours*. Random collections might be considered collectively responsible when aggregate individual behaviours – which are not coordinated and are not explained by individuals’ intention to produce a bad outcome – result in bad outcomes which could not have been produced by any individual alone (Sadler 2007, 488). The individual behaviours might consist either in aggregate actions or in aggregate inactions. Examples are the set of individuals who by driving every day cause global warming (a case of aggregate individual actions), and the set of people who choose not to be vaccinated thus contributing to the failure of a community to realize herd immunity (a case of aggregate individual inactions).

Now, one might argue that, in the case of random collections producing morally relevant outcomes through aggregate individual behaviours, the responsibility in question must be collective because individual members of random collections cannot be individually morally responsible. For example, one might appeal to the fact that any individual contribution is not sufficient to cause a bad collective outcome, such as global warming (Sinnott‐Armstrong 2005), or to the consideration that an individual contributory behaviour, such as driving a car, is not necessarily intrinsically immoral (Sadler 2007). However, it is doubtful that these types of considerations are enough to rule out individual responsibility and to warrant attribution of genuinely collective responsibility. Once again, these collectives are defined merely by the *aggregate* individual behaviours of certain individuals, i.e. by individuals acting (including failing to act) independently of one another without a common purpose and in unorganized ways, i.e. without forming any collective super‐entity. Thus, it is in principle possible to attribute moral responsibility to each individual without having to attribute moral responsibility to the collective. In particular, it is plausible to suppose that there are other moral reasons – different from the magnitude of one's contribution to the collective effect and from the intrinsic moral status of one's behaviour – for holding individual agents morally responsible for their contributory behaviours in a way that does not translate into attribution of responsibility to the collective.

For example, there might be considerations of fairness that impose on each individual the moral obligation to do their fair share to contribute to a desirable collective effect (e.g. the realization of herd immunity) or to contribute to the prevention of an undesirable collective effect (e.g. global warming); such considerations would then automatically render individuals morally responsible for having failed to make their fair contribution to a desirable outcome or to the prevention of an undesirable outcome. Once again, the identities, intentions and actions that are relevant for attribution of moral responsibility are merely the identities, intentions and actions of individuals: it is individuals acting independently of one another, and intending to act in a certain way, who fail to meet the relevant moral requirement, such as the requirements posed by considerations of fairness. There is no collective identity, collective intention or collective action that can explain the group's failure to realize or to prevent a morally relevant outcome and that are not reducible to an aggregation of individual identities, intentions and actions. Lacking such morally relevant properties at the collective level, the collective cannot be held genuinely collectively morally responsible for the relevant outcome or for the failure to prevent it. In other words, collectives identified by *aggregate* individual behaviours also fail to meet the first condition for attribution of genuinely collective moral responsibility.

### 3.5 Responsibility of collections engaged in joint actions

Sometimes collections are identified by the fact that individuals engage in joint actions that produce morally relevant outcomes. We argue that this is the only case in which collectives satisfy the two conditions for attribution of genuinely collective moral responsibility. Before showing this, let's examine what it means to engage in joint actions.

A joint action can be defined as a group action characterized by the following features: it requires the contribution of more than one individual for its performance; individuals do their part in bringing about a common end – or at least in bringing about different ends that “mesh” and are not inconsistent with each other (Bratman 1993; 1999); individuals intend to bring about this end or these consistent ends; they recognize that others are doing their part in bringing about such ends; they intend to make their contribution to this type of end because they recognize that others are doing the same; and they are all aware of all these conditions (Pettit and Schweikard 2006; Shockley 2007, 445).

The intentions involved in joint actions have been cashed out in terms of “collective intentions” (Isaacs 2006) or “we‐intentions” (Tuomela 2005; Ludwig 2016) or “shared intentions” (Bratman 1993; Sadler 2006). Of course, as Kirk Ludwig has suggested, these intentions can always be analysed in individualistic terms, in the sense that the intentions, including “we‐intentions”, are always ultimately attributed to single individuals, and not to the group as an independent entity (Ludwig 2016) – as is the case, for example, with corporate agents. However, there is an important sense in which these intentions can sometimes be characterized at the same time as genuinely “collective”, so that the individualistic analysis proposed by Ludwig does not conflict with the genuinely collective nature of the intentions in question. Let's see more in detail.

According to Michael Bratman's characterization, a shared intention to perform a joint action *J* is characterized as follows:

We intend to *J* if and only if (1) (*a*) I intend that we *J* and (*b*) you intend that we *J* (2) I intend that we *J* in accordance with and because of 1*a*, 1*b*, and meshing subplans of 1*a* and 1*b*; you intend that we *J* in accordance with and because of 1*a*, 1*b*, and meshing subplans of 1*a* and 1*b* (3) 1 and 2 are common knowledge between us (Bratman, 106).

On a different account of shared intentions provided by Brook Sadler, a group of individuals can be said to have a shared intention to perform a joint action when individual intentions to perform a certain individual action provide each other agent with a sufficient reason for intending to engage in a certain individual action (Sadler 2006, 124). Along similar lines, Margaret Gilbert has argued that, when individuals engage in a joint action, the wills of individuals are bound “simultaneously and interdependently”, i.e. in such a way that “each is (…) entitled to performance from the rest” (Gilbert 1990, 7–8).

For example, the group of people who collaborate to drag someone's automobile into a lake with the shared intention to drag the car into the lake participates in joint action (Mellema 2006). On Sadler's account of shared intention, this joint action is based on a shared intention in the sense that, for example, your individual intention to pull the car from the front towards the lake provides me with a sufficient reason for intending to push the car from the back towards the lake, and vice‐versa: this reciprocity defines the shared character of our intention and the joint character of our action.

For our purposes, the relevant questions are the following: is there a level of responsibility that can be said to be *genuinely* collective? In other words, can collections of individuals engaging in joint actions be held “collectively” morally responsible in a way that “does not fully distribute among individuals” (Isaacs 2006, 62) (as per our first condition for attribution of genuinely collective moral responsibility)? And can such collections at the same time be collectively morally responsible in a sense that is different from the individualistic sense of responsibility that is attributed to corporations, i.e. in a sense that accounts for the collective character of the collections (as per our second condition)? The answer to such questions would be affirmative if the collection of individuals engaging in joint action constituted a genuinely collective agent in those regards that are relevant for attribution of moral responsibility, i.e. an agent whose intentions, actions, and identity are genuinely collective. Let us then consider these three aspects in order.

As for the intentions, as said above, a joint action is one that is based on a “shared intention” (Bratman 1993), or “collective intention” (Isaacs 2006) or “we intention” (Tuomela 2005). It is possible to argue that a shared intention is not reducible to an aggregation of individual intentions to perform contributory actions, and that the notion of “shared intention” that characterizes joint actions can “support a meaningful concept of collective moral responsibility” (Isaacs 2006, 60). This is because, as both Bratman's and Sadler's definitions of shared intention suggest, the *relations* between individual intentions to engage in a joint action play an ineliminable role in defining the content of the shared intention: any single individual, considered in isolation, cannot have an intention with a shared content, and therefore a shared intention cannot be defined as just an aggregation of individual intentions. As put by Isaacs, “in so far as the relations are part of what constitutes a collective intention, no collective intention is fully reducible to the individual intentions that are components of it” (Isaacs 2006, 66). In Sadler's words, individual intentions are “normatively interlocked” (Sadler 2006). In this sense, although shared intentions can only be held by individuals and not by a super‐entity or a collective mind (Bratman 1993, 107), their character is not essentially individualistic, but substantially collective. More precisely, it is substantially collective because its *content* is substantially collective: a shared intention's content is determined by the *relations* among individual intentions, and therefore transcends the content of individual intentions. Accordingly, with regard to the intentions involved in the case of a collective engaged in joint action, the collective satisfies the first condition for attribution of genuinely collective responsibility: the collective has its own distinctive intention that does not boil down to a mere aggregation of individual intentions.

In setting out the conditions that must be satisfied for a group to be morally responsible in an intrinsically collective manner, we noted that the group must satisfy certain epistemic conditions. It must be able to intend, or at least foresee, the outcome or state of affairs for which it is appropriately held responsible. We suggest that insofar as a joint action is based on a shared (collective, or “we‐”) intention, this condition is often satisfied, too. The outcome will typically figure into the *content* of the intention. That is, the action will typically be based on a collective intention, for example to push the car into the lake. In fact, the goal of the joint action will typically need to be specified in the intention, in order for it to play the role of coordinating individual actions. Insofar as these intentions are normatively interlocked, they are substantially collective.

At least one prominent figure in debates over collective moral responsibility has recognized that the intentions of collectively responsible groups are normatively interlocked in the kind of fashion identified above (Miller 2006). However, Miller's account does not yield intrinsically collective moral responsibility as we understand the concept here, we maintain, because it does not satisfy our second condition for such moral responsibility. Collective agents engaged in joint actions (alone) can satisfy this condition. Since the intentions required for the existence of the collective's joint intention can only be held by the individuals who constitute the relevant collective, the shared intention of the collective is not the type of intention that might be attributed to a group agent like a corporation, i.e. an individualistic type of intention attributable to a particular type of super‐entity. On the contrary, each individual member of the collective shares the relevant intention or, in the terms we used above, the shared intention cannot but be the *product* of the intentions of individual members. Thus, the intentions are individual not with regard to their content, but with regard to the *subject* who holds them. It is necessary that each individual has a particular type of intention in order for the group to possess a certain shared intention. Accordingly, the shared intention of the collective satisfies also the second condition for attribution of genuinely collective moral responsibility whereby, to recall, the characteristics that are relevant for attribution of moral responsibility, in this case the intention of the collective, must be the product of the characteristics, e.g. the intentions, of its individual members.

We can therefore conclude that the shared intention of a group engaged in joint action satisfies both conditions for attribution of genuinely collective moral responsibility because, with regard to its *content*, the intention is not reducible to an aggregation of individual intentions, but is constituted by the relations among individual intentions (thus meeting the first condition); at the same time, with regard to the *subject* holding it, the shared intention can only be held by individuals each having their own individual intentions (thus meeting the second condition).

What about joint *actions* of the collective that are characterized by those intentions? We claim that they are also genuinely collective, and therefore, once again, they warrant attribution of genuinely collective moral responsibility. More particularly, unlike the behaviour of random collections, joint actions cannot be explained in terms of mere aggregation of individual behaviours, and therefore their collective character is not reducible to an aggregation of individual behaviours (Isaacs 2006, 64). For example, the joint action of a collective that drags a car into a lake is not defined simply in terms of aggregation of individual contributory actions; there is a distinctive collective dimension in the definition of the collective action that is not captured by mere aggregation. We can see this distinctive collective dimension of the joint action when we consider that we would not be able to explain what is happening when a group drags a car into a lake by simply describing the action each individual member of the group is performing; that would not be very informative. Rather, we need a description that refers to the level of group action and to the group as an agent. It is the *group* that drags the car into a lake, and the individuals are not acting as independent agents: they would not do what they do if their individual actions were not part of a joint action. A description that only referred to the level of individual actions would not capture this essential aspect. Because, in this sense, the joint action is not reducible to a mere aggregation of individual actions, with regard to the action performed the collective satisfies the first condition for attribution of genuinely collective moral responsibility.

At the same time, however, joint actions are not independent from the actions of individual members. Joint actions require individual agents to perform their *contributory* actions: without the contributions of a sufficiently large number of individuals, the group could not drag the car into the lake. Thus, because the collective action is the product of individual actions, it follows that, with regard to the action performed, the collective satisfies the second condition for attribution of genuinely collective moral responsibility: the collective can be said to perform an action – and can therefore be held responsible for an action – only because there are a number of individuals engaging in their individual contributory actions.

Finally, with regard to the identity of the group, there is an important sense in which groups that engage in joint actions can be considered genuinely collective agents, distinct from mere aggregations of individual agents (as was the case with some types of random collections), but also distinct from group agents conceived as special types of individual agents (as was the case with corporate agents). Let's examine these two aspects in order. A group that engages in a joint action is a group agent that exists, *qua* group, independently of the identity of its members, because it is defined by the (shared) intention to perform a certain action rather than by the fact that it is constituted by certain specific individuals or by a certain number of individuals: replace any one individual jointly dragging the car into the lake with any other individual with the same intention, or add or subtract individuals in a way that does not affect the group's intention and capacity to perform the action, and you still have the same collective agent identified by the shared intention to drag the car into the lake. As Gilbert writes, collectives engaged in joint actions constitute a distinct type of agent acting *as one body* (Gilbert 2006, 145). Because, exactly *like an acting, functioning body*, a collective engaging in a joint action is not reducible to a mere aggregation of individual identities – or, to stick to the metaphor, a mere aggregation of body parts – the collective that engages in a joint action meets in this respect the first condition for attribution of genuinely collective moral responsibility: the identity of the group is not a mere aggregation of individual identities. 6It is worth noting, although we do not have the space to analyse the debate here, that Gilbert's view here presented is controversial and has been criticized within the debate on collective action and collective responsibility, in particular by Miller and Makela (2005).


But, as far as identity is concerned, the collective agent engaged in joint action also satisfies the second condition for attribution of genuinely collective moral responsibility: it is an agent that can only exist because individual members exist, i.e. as a product of the existence of individual members (whoever they are), as the second condition requires. The identity of the collective agent is essentially plural because the group does not exist prior to and independently of the existence of individuals engaging in the joint action (whoever they are), as is instead the case with, for example, corporate agents. As put by Margaret Gilbert, “when a goal has a plural subject, (…) what is achieved is a binding together of a set of individual wills so as to constitute a single, ‘plural will’ dedicated to a particular goal” (Gilbert 1990, 7). The subject's identity is essentially “plural”, and therefore collective, in a way in which corporate agents’ identities are not, because, unlike the case of corporate agents, the existence of the entity engaging in joint action requires the existence of the individuals who contribute to the joint action: the collective only exists *because* such individuals exist and engage in the joint action. Thus, although not reducible to an aggregation of individual identities, the identity of the collective engaged in joint actions is in an important sense essentially plural: since the collective exists only because there are individuals engaging in joint action, its identity is in this sense the product of individual identities or, to put it differently, the collective exists as a group agent only because certain individuals exist and make their contribution to the joint action. Therefore, also with regard to its identity, the collection satisfies the second condition for attribution of collective responsibility.

## 4 Conclusion

Because in the case of collectives engaged in joint actions there is an agent that is genuinely collective, that has genuinely collective intentions, and that engages in genuinely collective actions, such collectives satisfy the two conditions for attribution of genuinely collective moral responsibility we presented at the beginning: the collective is responsible in a way that does not boil down to a mere aggregation of individual responsibilities – because the intentions, actions and identities of the collective are not reducible to an aggregation of individual intentions, actions and identities – but at the same time the collective is responsible in a way that accounts for the fact that the collective is constituted by a number of individuals – because the intentions of individuals are “normatively interlocked”, the action of the collective requires individuals to perform their contributory actions, and the subject that the individuals constitute is essentially plural.

Thus, in this paper we have argued that the collective of individuals engaging in joint actions is the only type of collective that can be held *genuinely collectively* morally responsible for the outcomes it causes, assuming that some form of moral responsibility can be attributed to this type of collective in the first place. *The authors’ work has been funded by the Oxford Martin School (OMS) at the University of Oxford, within its interdisciplinary project “Collective responsibility for infectious disease”.Alberto Giubilini's work has been funded by the Wellcome Centre for the Ethics and Humanities (WEH) at the University of Oxford through the Wellcome Centre Grant 203132/Z/16/Z.Neil Levy's work has been funded by the Wellcome Trust grant WT104848/Z/14/Z and the Australian Research Council DP110101810 “Challenges to Moral Responsibility”.We are very grateful to four anonymous reviewers for this journal for comments that enabled us to improve almost every aspect of this paper.

